# Gene Profiles in the Early Stage of Neuronal Differentiation of Mouse Bone Marrow Stromal Cells Induced by Basic Fibroblast Growth Factor

**DOI:** 10.1155/2020/8857057

**Published:** 2020-12-24

**Authors:** Lili Yu, Wei Hong, Haijie Yang, Yin Yan Xia, Zhiwei Feng

**Affiliations:** ^1^School of Basic Medical Sciences, Xinxiang Medical University, Xinxiang, China; ^2^Institute of Precision Medicine, Xinxiang Medical University, Xinxiang, China; ^3^Sanquan College of Xinxiang Medical University, Xinxiang, China; ^4^School of Life Science and Technology, Xinxiang Medical University, Xinxiang, China; ^5^School of Biological Science, Nanyang Technological University, Singapore

## Abstract

A stably established population of mouse bone marrow stromal cells (BMSCs) with self-renewal and multilineage differentiation potential was expanded *in vitro* for more than 50 passages. These cells express high levels of mesenchymal stem cell markers and can be differentiated into adipogenic, chondrogenic, and osteogenic lineages *in vitro*. Subjected to basic fibroblast growth factor (bFGF) treatment, a typical neuronal phenotype was induced in these cells, as supported by neuronal morphology, induction of neuronal markers, and relevant electrophysiological excitability. To identify the genes regulating neuronal differentiation, cDNA microarray analysis was conducted using mRNAs isolated from cells differentiated for different time periods (0, 4, 24, and 72 h) after bFGF treatment. Various expression patterns of neuronal genes were stimulated by bFGF. These gene profiles were shown to be involved in developmental, functional, and structural integration of the nervous system. The expression of representative genes stimulated by bFGF in each group was verified by RT-PCR. Amongst proneural genes, the mammalian *achate-schute* homolog 1 (Mash-1), a basic helix-loop-helix transcriptional factor, was further demonstrated to be significantly upregulated. Overexpression of Mash-1 in mouse BMSCs was shown to induce the expression of neuronal specific enolase (NSE) and terminal neuronal morphology, suggesting that Mash-1 plays an important role in the induction of neuronal differentiation of mouse BMSCs.

## 1. Introduction

Bone marrow stromal cells (BMSCs) contain mesenchymal stem cells [1] which are capable of differentiating into various progenies of mesodermal origins, such as osteoblast, chondrocyte, and adipocyte [2]. Studies have demonstrated that BMSCs can also exhibit neural phenotypes after transplantation and ameliorate the deficiency of different neuronal disorders [3–6]. BMSCs can be differentiated into various neural cells by adopting various protocols *in vitro* [7, 8], demonstrating the multipotent differentiation capacities and promising therapeutic values of BMSCs. However, the differentiation notion has been strongly debated recently as a stress response to the use of some chemicals adopted in neuronal differentiation protocols [9, 10]. It has also been suggested that BMSCs-derived neuronal cells only obtain partial neuronal features as assessed by the examination of a set of neuronal genes, compared to that obtained from neural stem cells [11]. Thus, further determination of neuronal traits of differentiated BMSCs with an appropriate differentiation strategy would be required for better illustration of this phenomenon albeit the presence of *in vivo* transplantation studies documenting the physiological properties of neuronal differentiated BMSCs.

Currently, most neuronal genes as examined in differentiated BMSCs were primarily used as benchmark markers for neuronal differentiation but seldom use for elucidating the mechanisms of initiating and/or regulating neuronal differentiation. Recent studies revealed that gene silencing to suppress the discordant phenotypes in BMSCs was also important for neuronal differentiation [12], suggesting that dynamic gene regulation was involved in neuronal differentiation of BMSCs. Moreover, it is rather difficult to determine the neuronal phenotypes of BMSCs based on the availability of a very limited pool of neuronal genes identified [11]. cDNA microarray analysis is a powerful tool for gene profiling analysis and has been widely used in gene expression kinetics determination. Two studies have applied this method to unravel the neuronal gene regulation profile in mouse BMSCs. For instance, Mori studied the neuronal differentiation of immortalized human BMSCs [13] and found that the genes analyzed were from late phase of one-week neuronal differentiation process, rather than from earlier initiating stage. This revelation would reflect the neural features of dominant genes in differentiated BMSCs. Yamaguchi, whom further studied the neuronal gene expression profile in DMSO induced neuronal differentiation of BMSCs [14], fell directly into the previous debate of BMSC differentiation as a direct result of the addition of certain compound which triggered an acute nonphysiological stress response thus rendering it unreliable. To study the neuronal differentiation under proper physiological condition, we have established a model of basic fibroblast growth factor (bFGF) induced mouse BMSC neuronal differentiation [15]. In this report, we intend to further examine the gene expression profile at early stages of neuronal differentiation as induced by bFGF with microarray analysis in our attempt to further clarify the mechanisms underlying neuronal differentiation of BMSCs.

bFGF, a 154-amino acid peptide, is the first growth factor employed in pilot study examining the neuronal differentiation capacity of BMSCs [16, 17]. Besides its wide range of biological activities on cell growth, differentiation, and survival, FGF evoked signaling pathway has also been shown to be the prerequisite factor for neural induction in chick embryo [18] and inducer of posterior neuronal precursors in the neural tube [19]. In the nervous system, members of the FGF affect the differentiation and migration of neurons, the formation and maturation of synapses, and the repair of neuronal circuits following insults [20]. Basic fibroblast growth factor increases the transplantation-mediated therapeutic effect of bone mesenchymal stem cells following traumatic brain injury [21]. In *in vitro* studies, bFGF could induce neurotube formation from ES cells [22], and transdifferentiation of the pigmented epithelial cell into retinal neurons [23] has also been reported. Dual delivery of bFGF- and NGF-binding coacervate confers neuroprotection by promoting neuronal proliferation [24]. All these findings singled out bFGF as a potent proneural factor with wide range capabilities. However, the mechanisms underlying, especially that of neuronal transdifferentiation, are poorly understood. BMSCs transfected with the intracellular domain of the Notch was found to be more effectively induced into neuron-like cells in the presence of bFGF [9]. The Notch signaling pathway has been demonstrated to play a pivotal role in neurogenesis of *Drosophila* [25]. Deregulation of Notch signaling is involved in many neurodegenerative diseases and brain disorders [26, 27]. Several groups of molecules, including the bHLH family, are directly regulated by Notch pathway, which conventionally regulate neural determination and differentiation [28, 29]. Furthermore, all genes of the Notch pathway are found to be preserved in vertebrates [30]. A clear example would be Mash-1, a bHLH family member gene, which is obviously affected in mouse by the mutation of Notch signaling pathway and in which contributed to neurogenesis [31]. However, it remains unclear how Notch signaling is involved in regulating the neuronal differentiation of BMSCs.

In our attempt to better understand the genes involved in the neuronal differentiation of BMSCs, we adopted the cDNA microarray strategy to sequentially analyze bFGF-activated neural genes at various time points. We observed that bFGF-activated neural genes exhibit distinct expression kinetics and most importantly are involved in various neural activities. We further demonstrated that the mammalian proneural gene, Mash-1, was induced at an early stage of differentiation, and BMSCs can be driven into neuronal cells by the overexpression of Mash-1. Our work provides an insight of the molecular mechanisms of neuronal differentiation of BMSCs by bFGF.

## 2. Material and Methods

### 2.1. Isolation and Culture of Mouse BMSCs

Bone marrow stromal cells (BMSCs) of 8-week-old male Swiss mice were initially cultivated in Dulbecco's Modified Eagle Medium (DMEM; Invitrogen) supplemented with 10% fetal bovine serum (FBS; Invitrogen), 10% newborn calf serum (NCF; Invitrogen), 100 U/ml penicillin, and 100 mg/ml streptomycin (Invitrogen). The cells were incubated at 37°C in 5% CO_2_ in 100 mm tissue culture dishes for 2-3 days, and nonadherent cells were removed by medium replacement. Adherent cells were further cultured for 7-10 days to reach 90% confluence and defined as the first passage. After detachment with 0.25% Trypsin/0.5 mM EDTA, around 25% of the cells were transferred to a new culture flask. A fairly homogeneous cell population was achieved after 4-5 passages, and these cells could be continuously cultured for up to 50 passages without obvious growth retardation.

### 2.2. Fluorescence Activated Cell Sorting (FACS) Analysis

Samples containing 1 × 10^6^ cells were resuspended in 300 *μ*l of FACS buffer (PBS + 2%FBS) and incubated for 20 minutes at 4°C with 3 *μ*l of fluorescein isothiocyanate- (FITC-), phycoerythrin- (PE-), allophycocyanin- (APC-), and peridinin chlorophyll protein- (Per-CP-) conjugated antibodies, respectively, against surface markers CD34, CD44, CD45, CD90.1, CD117, CD105, and Sca-1. All antibodies were from Pharmingen (BD Pharmingen). After addition of 6 ml FACS buffer, cells were pelleted by 5 min centrifugation at 260 × g at 4°C. The labelled cells were resuspended in 500 *μ*l FACS buffer and analyzed by BD FACS Aria flow cytometer.

### 2.3. Adipogenic Differentiation

Flat BMSCs were plated onto 6-well tissue culture plates at a density of 5 × 10^4^ cells per well and incubated for 24 hours before treatment with adipogenic differentiation medium (500 nM Dexamethasone, 250 *μ*M isobutyl-methylxanthine (IBMX), 100 *μ*M indomethacine, and 5 *μ*M insulin in basal DMEM supplemented with 10% FBS, 10% FCS, and 1% penicillin-streptomycin). The media was changed every 3 days, and adipogenic differentiation was assessed by Sudan Black staining 10 days postinitial adipogenic induction.

### 2.4. Sudan Black Staining

Cells were rinsed with PBS and fixed with Baker's Solution for 10 minutes, followed by 3 minutes exposure to 100% propylene glycol. The cells were then stained with Sudan Black for 2 hours followed by 3 washes with water. This was followed by another 2 minutes of exposure to 85% propylene glycol and a final 3 washes with water. Positive Sudan Black stained cells appear blue black under light microscopy.

### 2.5. Chondrogenic Differentiation

Chondrogenic differentiation was induced using the high-density micromass culture technique [32]. A 10 *μ*l drop of a concentrated cell suspension (1 × 10^6^ cells/ml) was placed onto the centre of the well (of a 24-wells tissue culture plate) and allowed to attach and aggregate for 2 hours at 37°C with 5% CO_2_. Chondrogenic induction medium (50 nM ascorbic acid-2-phosphate, 6.25 *μ*g/ml insulin, and 10 ng/ml transforming growth factor *β*1 in basal DMEM supplemented with 1% FBS and 1% penicillin-streptomycin) was then gently overlaid, avoiding disruption to the attached cells. The media was then changed every 3 days, and the chondrogenic phenotype was determined by Alcian Blue staining 3 weeks postinitial chondrogenic induction.

### 2.6. Alcian Blue Staining

Micromass cultures were rinsed with PBS and fixed in 2% paraformaldehyde for 15 minutes. They were then rinsed with 3% acetic acid for 5 minutes to bring down the pH to 2.6 followed by 20 minutes staining with 1% Alcian Blue (pH 2.6). Cells were then washed twice with water before being observed under light microscopy. The highly sulfated proteoglycans of cartilage matrices were positively stained blue.

### 2.7. Osteogenic Differentiation

1 × 10^4^ cells were plated onto a 24-wells plate and cultured in an osteogenic differentiation medium (50 *μ*M ascorbic acid-2-phosphate, 10 mM *β*-glycerophosphate, and 100 nM dexamethasone in DMEM basal medium supplemented with 10% FBS and 1% penicillin-streptomycin) after 24 hours incubation. The media was changed every 3 days. Osteogenic differentiation was assessed by Alizarin Red S staining 3 weeks postinitial osteogenic induction.

### 2.8. Alizarin Red S Staining

Cells were rinsed twice with PBS and fixed in ice-cold 70% ethanol for an hour at –20°C. After a few rinses with deionised water, cells were incubated with 40 mM Alizarin Red S (pH 4.1) for 20 minutes at room temperature with gentle shaking. A further 4 washes with deionised water were carried out to clear away unspecific staining on the cells. Secretion of calcified extracellular matrix was confirmed as red stains with Alizarin Red S Staining.

### 2.9. Neurogenic Differentiation

For neuronal differentiation, BMSC cells were isolated and split every other day. Neurogenic differentiation was induced by exposure to 10 ng/ml basic fibroblast growth factor. The neuronal differentiation medium (low glucose DMEM supplemented with 10% of FBS and 10 ng/mL bFGF) was added starting from passage 5. Neuronal differentiation was monitored by microscopic observation, and the expression of neuronal markers was analyzed by RT-PCR, western blotting, and immunohistochemistry.

### 2.10. cDNA Microarray Analysis

BMSCs were harvested at 4, 24, and 72 hours post-bFGF treatment, and total RNAs were extracted using Trizol reagent following the manufacturer's instructions (Invitrogen). cDNAs were synthesized and subjected to Agilent's Mouse Development Oligo Microarray (Agilent Technologies). The microarray experiment was repeated twice using RNA samples from independent experiments, and the result reported was based on the average of these two independent experiments. cDNAs which showed at least 2-fold difference were selected for further analysis. cDNA microarray experiment was blinded to investigator, and microarray data analysis was also assessed by three independent investigators who were blinded to the sample.

### 2.11. Reverse Transcription Polymerase Chain Reaction Analysis

The expression of ChAT, GAP43, Hes5, Mash1, NSE, Tau, SERT, MAP2, and *β*3-tubulin was determined by reverse transcription polymerase chain reaction (RT-PCR). Total cellular RNA was isolated using Trizol reagent (Invitrogen) and reverse transcribed using Invitrogen's First Strand cDNA Synthesis Superscript III RT Kit. PCR amplification was performed using the following primers (shown in brackets in the following order: sense and antisense), ChAT (5′-TGTGAGGAGGTGCTGGACTTA-3′, 5′-GCCAGGCGGTTGTTTAGATA-3′), GAP43 (5′-GCTAGCTTCCGTGGACACATA-3′, 5′-AGGCACATCGGCTTGTTTA-3′), Hes5 (5′-ATGCTCAGTCCCAAGGAGAA-3′, 5′-CGCTGGAAGTGGTAAAGCAG-3′), Mash1 (5′-GCTCTGGCAAGATGGAGAGT-3′, 5′-CCAGGTTGACCAACTTGACC-3′), Tau (5′-CCCTGGAGGAGGGAATAAGA-3′, 5′-GCAGACACTTCATCGGCTAGT-3′), and NSE (5′-TGATCTTGTCGTCGGACTGTGT-3′, 5′-CTTCGCCAGACGTTCAGATCT-3′). All primer sequences were determined through established GenBank sequences. Duplicate PCRs were amplified using *β*-actin (5′-TGTTACCAACTGGGACGACA-3′, 5′-TCTCAGCTGTGGTGGTGAAG-3′, 392 bp) as a control for assessing PCR efficiency and subsequent analysis by agarose gel electrophoresis.

### 2.12. Quantitative Real-Time PCR (qRT-PCR)

Total RNA was extracted using Trizol reagent in accordance with the manufacturer's instructions. After the treatment with RNase-free DNase I (Invitrogen) to remove genomic DNA contamination, RNA (1 *μ*g) was reverse transcribed into cDNA using Moloney murine leukemia virus reverse transcriptase (Promega, Madison, USA). Real-time PCR was performed using Power SYBR Green PCR Master Mix (Applied Biosystems, Foster City, CA, USA) in an ABI PRISM 7500 real-time cycler (Applied Biosystems). The mRNA levels of the target gene were normalized to that of *β*-actin. Melting curves were constructed using the Dissociation Curves software to ensure that only a single product was amplified. According to the method of Livak and Schmittgen, the intensity of the relative expression was based on 2 − *ΔΔ*CT equation. The sequences of primers for PCR are listed as follows: SERT (5′-TGCCTTTTATATCGCCTCCTAC-3′, 5′-CAGTTGCCAGTGTTCCAAGA-3′), MAP2 (5′-TCAGGAGACAGGGAGGAGAA-3′, 5′-GTGTGGAGGTGCCACTTTTT-3′), *β*3-tubulin (5′-CATGGACAGTGTTCGGTCTG-3′, 5′-CGCACGACATCTAGGACTGA-3′), SV2a (5′-GGTTCACGACACCAACATGC-3′, 5′-ACTTTGGTTCGGGCTGCATA-3′), and SNAP-25 (5′-CGCAATGAGCTGGAGGAGAT-3′, 5′-TCCCTTCCTCAATGCGTTCC).

### 2.13. Western Blot

Cells were lysed with lysis buffer (50 mM Tris-Base, 100 mM NaCl, 5 mM EDTA, 1 mM EGTA, 5 mM MgCl_2_, 10% glycerol, 1% Triton X-100, and Roche's Complete Protease Inhibitor tablet) and were centrifuged at 14,000 × g for 20 minutes at 4°C. 50 *μ*g of proteins from the supernatants was separated by 8%-12% SDS-PAGE gel and transferred to a PVDF membrane. Immunoblotting was carried out with polyclonal antibodies to ChAT, GAP43, Hes5, Mash1, NSE, Tau, and *β*-actin, respectively. After incubation with peroxidase-conjugated secondary anti-immunoglobulin antibodies, the membranes were developed using Pierce's West Pico Chemiluminescence method.

### 2.14. Electrophysiology

For whole-cell current recording, cells were patched as previously described (37). The bath solution contained (in mM) NaCl 150, KCl 5, MgCl_2_ 1, CaCl_2_ 2.2, Hepes 10, and pH 7.3 with NaOH, and the osmolarity was adjusted to 310 mOSM with glucose. The pipette solution contained (in mM) aspartic acid potassium salt 120, MgCl_2_ 5, EGTA 0.5, Hepes 10, ATP 2, GTP 0.3, and pH 7.3 with KOH, and the osmolarity was adjusted to 295 mOSM. Whole-cell current was obtained under voltage clamp with Axopatch 200B amplifier (Axon Instruments); the software used was pClamp 8.1 (Axon Instruments). The current was sampled at 10 kHz and filtered at 1 kHz. Leak was subtracted on-line using P/4 protocol. The pipette used was 1-2 m*Ω*, and the series resistance was typically <5 m*Ω* and compensated by 75%. The holding potential was set at –70 mV and depolarized to different voltages to evoke channel opening.

### 2.15. Plasmid Construction

The coding region of mouse Mash-1 was cloned into the pREP10 vector using convenient restriction sites. PCR product of Mash1 was digested with *Kpn* I and *Xho* I, purified and ligated to digested pREP10 expression vector. The bHLH domain of Mash-1 was then fused to the V5 epitope. All constructs generated by PCR were verified by sequencing prior to use, and protein expression was confirmed by western blotting with antibodies to Mash-1 and epitope tag V5.

### 2.16. Transfection

5 × 10^5^ cells were seeded to 100 mm tissue culture dishes and allowed to grow overnight prior to transfection. 30 *μ*g of plasmid DNA was mixed with 60 *μ*l of Lipofectamine 2000 Reagent to initiate the complex forming. Transfection was initiated by adding the mixture to the cells directly. Cells were extracted and assayed 3 days posttransfection.

### 2.17. Immunofluorescence Staining

For immunofluorescence staining, cells were seeded on Lab-Tek chamber slides and fixed with precold methanol at –20°C for 3 min. Then, the cells were permeabilized with 0.2% TritonX-100 in PBS for 15 min and blocked with 10% normal goat serum in PBS at room temperature for 30 min. The cells were incubated with the primary antibodies at 37°C for 1 h, washed with PBS twice, and then were incubated with the appropriate fluorescein isothiocyanate-conjugated secondary antibodies for 30 min. Finally, the cells were mounted with an antifluorescence quenching sealing liquid for observation under a fluorescence microscope.

### 2.18. Statistical Analysis

All data are presented as mean ± standard error of the mean (SEM). Student *t*-test was used to determine significance between individual comparisons. One-way ANOVA tests with Bonferroni's corrections were used for multiple comparisons. The calculations were performed using the SPSS version 11.0 statistic software. *P* values < 0.05 were considered statistically significant.

## 3. Results

### 3.1. Multipotency of BMSCs at Different Passages

**Figure 1:**
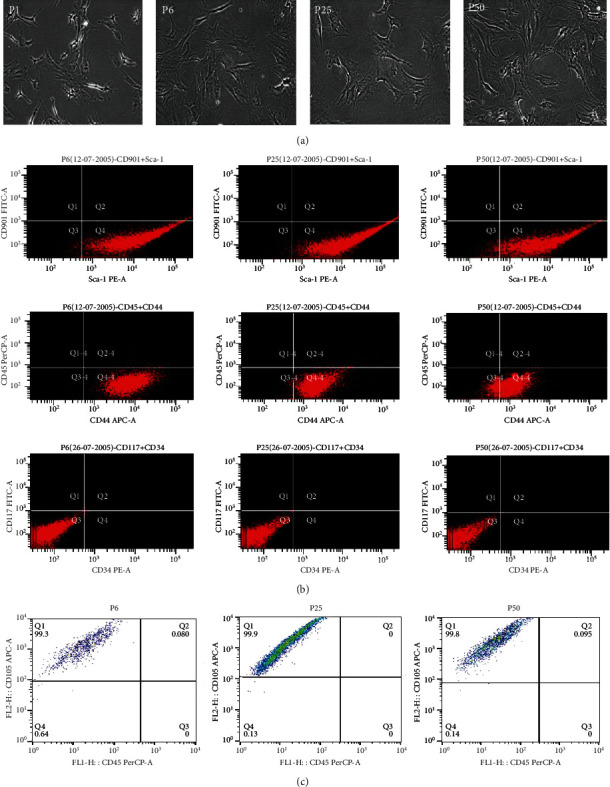
Characterization of BMSCs. (a) BMSCs from initial isolation to passage 50. Cell morphology became relatively homogeneous from passage 6 onwards and maintained the appearances till passage 50 or later. (b) Fluorescent-activated cell sorting (FACS) analysis for cell surface markers. Throughout the passages, markers for mesenchymal stem cells, Sca-1 and CD44, remained highly elevated and slightly decreased with passage number. The expressions for CD90.1, CD45, CD117, and CD34 were negligible throughout thus excluding contamination of hematopoietic stem cells from the isolated culture. (c) FACS analysis for CD105 and CD45. The expression of CD105 was positive, and CD45 was negligible.

Mouse BMSCs were isolated from the femur bones of mice and cultured in DMEM supplemented with 10% FBS, 10% FCS, and 1% penicillin-streptomycin. A fibroblast-like cell population eventually was obtained after 4-5 passages, and this cell population can grow continuously for more than 50 passages under our experimental condition without losing much of its plasticity and appeared healthy. Cell growth rate was lower at early passages due to competition from other cell types, and a stable stage of homogenous cell population was reached after 4-5 passages. Cell morphology as tracked from passages 6 to 50 showed a consistent phenotype and level of homogeneity (Figure 1(a)). It has been reported that cell features and plasticity may vary for the long-term *in vitro* culture [33]. Thus, we examined several cell surface markers of BMSCs at different passages. The flow cytometry results showed that these cells exhibited high levels of Sca-1 [34], a stem cell marker, and CD44 [35], a mesenchymal marker across passages 6, 25, and 50 (Figure 1(b)). As suspected, all hematopoietic cell markers, CD90.1, CD45, CD34, and CD117, were undetectable or poorly expressed in BMSCs at all passages (Figure 1(b)). We also detected the other marker CD105 for identification of BMSCs at different passage, which was positive as suspected (Figure 1(c)). These data demonstrated that this highly homogeneous cell population at indicated passages exhibit mesenchymal stem cell markers and is free from obvious hematopoietic cells contamination.

**Figure 2:**
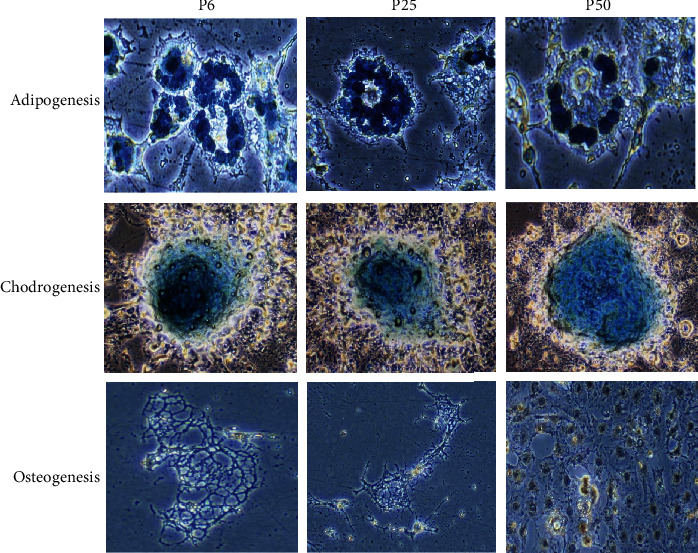
Developmental potential ability of BMSCs in vitro. BMSCs at passages 6, 25, and 50 were induced under adipogenic, chondrogenic, and osteogenic conditions. The presence of clusters of lipid containing adipocytes was detected by Sudan blue staining after adipogenic induction for 5 days. Cells show chondrocyte morphology and are positive for Alcian black stain after chondrogenic induction for 21 days. Cells show osteocyte morphology and are positive for Alizarin red stain after osteogenic induction for 21 days.

To determine the multilineage differentiation capacity of BMSCs, cells were subjected to a series of differentiation towards mesenchymal origins, namely, adipocytes, chondrocytes, and osteoblasts. As shown in Figure 2, cells at 6 and 25 passages could differentiate into adipocytes and chondrocytes as determined by biochemical tests, while cells at passage 50 could differentiate into all three cell types. These results are consistent with earlier observation that BMSCs with the capacity of adipocyte differentiation have high capacity of colony forming [36] and probably a longer life span and plasticity as well. Though the default commitment of BMSCs is to undergo osteogenic differentiation, this phenomenon is only observed at the early developmental stages of BMSCs.

### 3.2. Neuronal Differentiation of BMSCs Induced by bFGF

**Figure 3:**
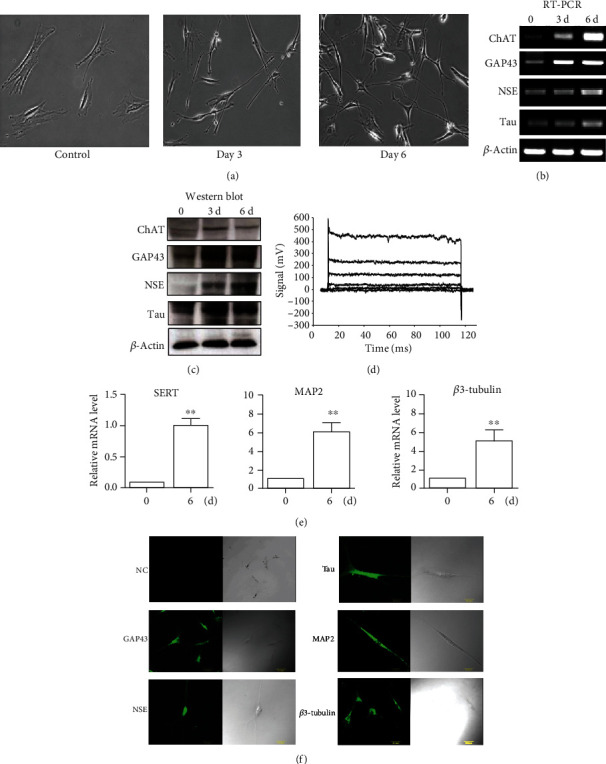
Neuronal differentiation induced by bFGF. (a) Morphological observation of BMSCs subjected to 10 ng/ml bFGF for 3 and 6 days. Typical signs of a developing neuron, such as cell body shrinkage, axonal elongation, and neurite outgrowth, were observed. The distinctive observation is heightened at day 6 postadministration. (b) RT-PCR representation of marked increase in genes regulating neuronal development and maturation as evidently shown by increase of ChAT, GAP43, NSE, and Tau. (c) Western blotting results supported the same observation of increased neuronal gene expression at protein levels which strengthened the deduction of a neuronal development and eventual maturation. (d) The electrophysiological properties of these differentiated cells were examined by patch clamping method at day 6 post-bFGF administration. (e) The expressions of SERT, MAP2, and *β*-tubulin were determined by qRT-PCR at day 6 post-bFGF administration. Data of qRT-PCR indicate the mean ± SEM of three experiments. (f) Distribution of neuronal differentiation markers. Immunofluorescence staining using specific antibodies against GAP43, NSE, Tau, MAP2, and *β*3-tubulin was performed in BMSCs with bFGF treatment for 3 days. NC: negative control. Images are representatives of at least three experiments.

A model for mouse BMSC neuronal differentiation by bFGF [15] has been established to elucidate the molecular mechanism of neuronal differentiation of mouse BMSCs in physiological conditions. Mouse BMSCs treated with bFGF alone for 3 to 6 days exhibited typical neuronal morphology as evidenced by shrunken cell bodies and elongated cell processes (Figure 3(a)). Choline O-Acetyltransferase (ChAT) is the enzyme responsible for the biosynthesis of acetylcholine, which presently the most specific marker for identifying cholinergic neurons in the central and peripheral nervous systems. Growth associated protein (GAP) 43 is a polypeptide that is induced in neurons when they grow axons. Neuron-specific enolase (NSE) is a dimeric isoform of the glycolytic enzyme enolase found mainly in neurons, which is relatively specific for neuronal cells. A Tau protein is a protein found in neurons, primarily in the central nervous system, which interacts with tubulin to strengthen the neural tubes in the axons of neurons. In order to gather more evidences on neuronal phenotypes, we examined these neuronal differentiation markers by RT-PCR. As shown in Figure 3(b), mRNA levels for ChAT, GAP-43, NSE, and Tau were significantly upregulated upon bFGF treatment. Similar expression patterns in protein levels were also observed for these genes (Figure 3(c)). Full unedited western blot images are supplied in Supplemental data 1. Consistently, after bFGF treatment, differentiated neuronal cells exhibited characteristic electrophysiological properties as determined by whole-cell patch clamp technique. As shown in Figure 3(d), the typical whole cell current trace was detected in 6-day neuronal differentiated cells. We also detected other related markers. Serotonin transporter (SERT) is a critical serotonin transporter in the function of serotoninergic neurons, as a biomarker of serotonergic neurotransmission in the central nervous system. Microtubule-associated protein (MAP) 2 is thought to be involved in microtubule assembly, which is an essential step in neurogenesis. *β*3-Tubulin is one of six *β*-tubulin isoforms, which is highly expressed during axon guidance and maturation and that is a great neuronal marker. To determine these markers, qRT-PCR method was employed, and a high level of SERT, MAP2, and *β*3-tubulin expression was detected (Figure 3(e)). We also used immunofluorescence staining to test these proteins, and results showed that BMSCs treated with bFGF exhibited high expression of neurogenesis-related markers, including GAP43, NSE, Tau, MAP2, and *β*3-tubulin (Figure 3(f)). As our data indicated in these figures, we found both ChAT and SERT were induced in bFGF-induced BMSCs, which showed these cells have a potential ability towards cholinergic and neurons serotoninergic neuronal differentiation. All this data indicates after bFGF treatment, BMSCs differentiate towards at neuronal direction.

### 3.3. Overview of bFGF-Induced Gene Expression Profiles in Mouse BMSCs

**Figure 4:**
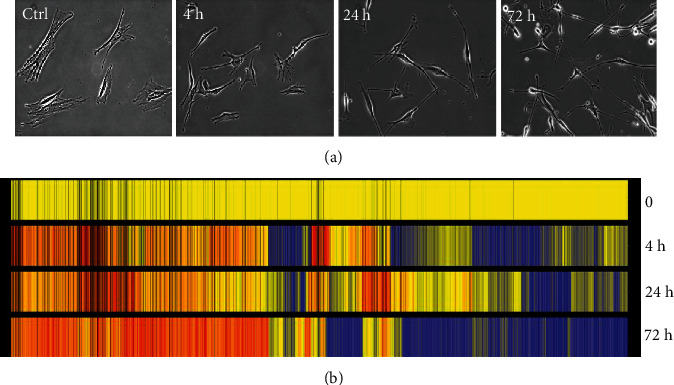
Cluster analysis of genes associated with neuronal differentiation. Colored bar representation of the cDNA microarray using total RNAs extracted from BMSCs at 4, 24, and 72 hours post-bFGF treatment. The yellow bars represent the levels of gene in the untreated cells, and the red ones are the upregulated genes, whereas the blue bars stand for the downregulated genes.

The gene expression profiles of mouse BMSCs were analyzed by microarray analysis in attempt to gather a more complete picture of genes participating in neuronal differentiation and in determining the molecules responsible for converting BMSCs into neuronal cells. Since we have previously observed that neuronal phenotypes can be strongly induced 72 hours post-bFGF treatment [15], total RNAs were extracted from BMSCs after 0, 4, 24, and 72 hours treatment by bFGF, respectively, which was supposedly representing the neural initiation, differentiation, and maturation stages of these differentiated cell phenotype. Otherwise, the fibroblast-like BMSCs started to change its morphology to that resembling neuronal cell phenotype at around 24 hours posttreatment (Figure 4(a)), and these changes were tracked further to include observation of neurite-like structures outgrowth and even to a point of joining and connecting with adjacent differentiated cells at 72 hours, suggesting that the time points designed were suitable for detecting early expressed genes. Cross-talking of adjacent cells mimics the action of matured neurons in forming synapses for information transfer and having been able to observe such a connection in our differentiated neuronal cells do provide evidences with a glimpse of hope that these differentiated cells might eventually become functional neurons. After reverse-transcription into cDNA, samples were hybridized with DNA oligonucleotide chips representing around 21,000 genes (Agilent Technologies). To ensure the results, two independent neuronal differentiation experiments and microarray analysis were performed. From the even gene expression values at different time points, 1148 genes were identified whose expression levels were more than two-fold changes at one or more time points compared with that of untreated sample. These genes were grouped according to their expression pattern with hierarchical clustering and shown by colored bar representation (Figure 4(b)).

**Figure 5:**
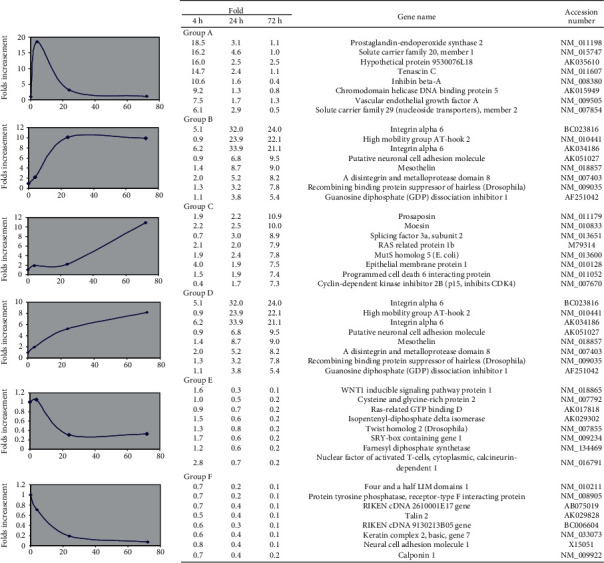
Classification of differentially expressed genes. Classification of top upregulated genes with reference to various neural stage activities analyzed by cDNA microarray analysis on bFGF-treated BMSCs for 4, 24, and 72 hours. The values at each time points were obtained by comparing with the level of the untreated. Several typical genes in neuronal developmental function at different six groups after bFGF treatment were listed.

As stated above, mouse BMSCs started to exhibit neuronal morphology at 24 hours post-bFGF treatment, and we assume that the selected time points could reflect the neuronal induction, differentiation, and maturation of BMSCs at 0, 4, 24, and 72 hours of bFGF treatments, respectively. Several typical genes in each group were listed with the concrete expression ratio at different time point compared to untreated cells (Figure 5). Accordingly, changes in gene expression were grouped as early upregulated (Figure 5, Group A), middle upregulated (Figure 5, Group B), late upregulated (Figure 5, Group C), and constitutively upregulated (Figure 5, Group D) based on the folds of induction at these time points. Since the gene downregulation was also shown to be involved in the neuronal differentiation of BMSCs [12], two downregulated groups were also listed, the middle downregulated (Figure 5, Group E) and constitutively downregulated (Figure 5, Group F) gene expressions.

### 3.4. Neuronal Gene Expression Profiles

From the list of genes upregulated by bFGF, about 300 neural-related genes with at least 5-fold upregulation at one or more time point were identified. These genes can be classified into two groups, early upregulated neural genes and continuous upregulated neural genes (Table 1). There are mainly four genes in the first group, synaptic vesicle glycoprotein 2a (SV2a), chemokine (C-C motif) receptor 1 (CCR1), synaptosomal-associated protein 25 (SNAP-25), and gamma-aminobutyric acid (GABA) receptor. SV2a has been demonstrated to be involved in vesicle trafficking and exocytosis, processes crucial for neurotransmission, and plays a crucial role in modulating epileptogenesis [37]. CCR1-mediated signal transduction is critical for the recruitment of effector immune cells to cause neuroinflammation and is an early and specific marker of Alzheimer's disease [38]. SNAP-25 is one of the key proteins involved in the formation of neural soluble N-ethylmaleimide-sensitive factor attachment protein receptors (SNAREs), which are responsible for the calcium-dependent exocytosis of neurotransmitters, that is a major step in neurotransmission and normal functioning of brain [39]. GABA receptors are involved in inhibitory synapses within the central nervous system [40]. There are mainly four genes in the continuous upregulated neural genes group, including semaphorin 5A, P2-activated kinase 1, putative neuronal cell adhesion molecule, and semaphoring 6A. Semaphorin 5A is a bifunctional axon guidance cue for axial motoneurons [41]. Putative cell adhesion membrane molecule Vstm5 regulates neuronal morphology and migration in the central nervous system [42]. Nrf2 in ischemic neurons promotes retinal vascular regeneration through regulation of semaphorin 6A [43].

Similarly analyses as above, about 300 neural-related genes with at least 2-fold upregulation at one or more time point were also identified. These genes can be classified into several groups according to likelihood of neural functions involved (Table 2). The first group participates in neuronal development, and the representative genes are fibroblast growth factor receptor (FGFR), Nestin, Chordin, and the empty spiracles homolog 1. Nestin is a widely used marker for neuronal stem cell and expressed at early stage of neuronal differentiation [16, 44]. Chordin, a homolog of short-gastrulation in *Drosophila*, has been shown to stimulate neural induction in Xenopus [45]. FGFR was demonstrated to play an important role in neurogenesis [46], and we also found that FGFR-1 is required for the neuronal differentiation of BMSCs induced by bFGF [15]. Genes in the second group are proneural transcription factors [47], such as achaete-scute complex homolog-like 1 (Mash-1), hairy and enhance of split 5 (Hes-5), neuronal PAS domain-2 and -3, and neuronal D4 (neurogenic differentiation 4). Mash-1, also known as achaete-scute family bHLH transcription factor 1 (ASCL1), is regulated by Notch signaling [29]. Mash-1 functions in controlling the generation of progenitors in central nervous system (CNS) and external sensory organs in the peripheral nervous system (PNS) [48, 49]. This gene encodes a member of the bHLH family of transcription factors. The protein activates transcription by binding to the E box (5′-CANNTG-3′). Dimerization with other bHLH proteins is required for efficient DNA binding. This protein plays a role in the neuronal commitment and differentiation and in the generation of olfactory and autonomic neurons [50]. Interestingly, Hes-5 gene was shown to suppress the function of Mash family and promote cell proliferation via the Notch signaling [51, 52]. Neuronal D4, a member of NeuroD family, plays a neuronal determination effect in neural development, and overexpression of Neurogenin1 can induce neuronal-like phenotype in human mesenchymal stem cells [53, 54]. Neuronal PAS domain proteins are shown to be involved in bFGF-stimulated adult neurogenesis [55]. In conclusion, gene upregulation from these two groups suggests that they may be involved in neurogenesis of BMSCs. The third group represents axonal, which includes genes such as Dynein-4 and -10, and Netrin-1 is involved in axonal genesis. Dynein family factors are the key motors of axon growth [56]. Netrin is a potent axon growth factor [57] and has been observed to be induced more than 20 folds in this study. Overall, these groups of genes may contribute to the cell morphological changes as reported here.

Genes involving neural activities or functions can be grouped into either neurotransmitter molecules (the forth group) or ion transport (the fifth group) or peptide signaling (Table 2). A typical molecule of neurotransmitters is Complexin-1, which is a cytosolic molecule and can bind to SNAP receptor to compete with SNAP [58]. Two syntaxin family members, Syntaxin 3 and 4A, were also upregulated in this study. Syntaxin family members are expressed only in the nervous system and implicated in docking of synaptic vesicles [59]. The highest expressed molecule in ion transport is Gamma-aminobutyric acid receptor alpha 6. Genes in this family play a very important role in depolarization of neuronal cells [60]. Several members of semaphorin were upregulated by bFGF in this study. Semaphorin is expressed on the cell membrane and has been demonstrated to regulate guidance of growth corns [61].

A group of receptor genes was also clearly upregulated, such as G-protein-coupled receptors (the sixth group) (Table 2). G-protein-coupled receptors were also demonstrated to have nonneuronal functions. GRP54, for instance, was involved in reproductive development [62] and GRP65 in signaling cascade [63]. Additionally, the opioid receptor is well known for mediating the morphine action [64].

### 3.5. Mash-1 Promotes BMSCs Neuronal Differentiation

Neuronal differentiation involves complicated gene regulation, in which the molecules related to signal transduction and gene transcription play a crucial role. We thus would like to suggest the following neuronal differentiation model complete with gene regulation networks after taking into consideration our findings on neuronal genes as induced by bFGF and the neuronal differentiation process. In determining the gene pool which plays a major role in turning BMSCs into neuronal cells, we decided to focus on genes from the first two classes, which are involved in neurogenesis and are also related to gene transcription. Amongst proneural genes, the mammalian achate-schute homolog 1 (Mash-1), a basic helix-loop-helix transcriptional factor, was further demonstrated to be significantly upregulated. Mash-1 stood out as it was upregulated more than 4 folds at 4 hours and reached another 8 folds at 72 hours posttreatment, respectively (Table 2).

**Figure 6:**
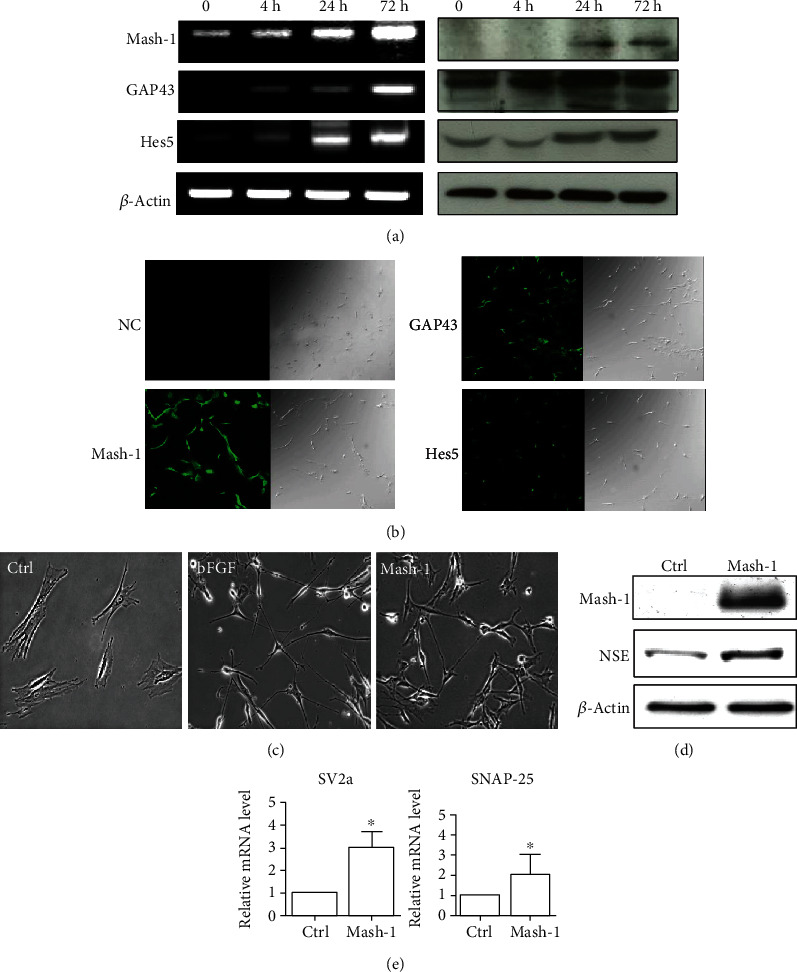
Overexpression of Mash-1 promotes neuronal differentiation of BMSCs. (a) RT-PCR (left) and western blot (right) representation of selected markers from cDNA microarray which showed consistencies with the data as analyzed. (b) Distribution of neuronal differentiation markers. Immunofluorescence staining using specific antibodies against GAP43, Mash-1, and Hes5 was performed in BMSCs with bFGF treatment for 24 hours. NC: negative control. Images are representatives of at least three experiments. (c) Mash-1 overexpressing BMSCs showed a typical neuronal morphology. (d) Western blotting analysis showed a significant increase of the NSE expression in Mash-1 overexpressing BMSCs compared to control cells. (e) The expression of SV2a and SNAP-25 were determined by qRT-PCR in Mash-1 overexpressing BMSCs. Data of RT-PCR indicate the mean ± SEM of three experiments.

RT-PCR analysis for Mash-1 was carried out to assess the credibility of our microarray result at the mRNA level. This step is to ensure that the result obtained from cDNA microarray is not due to artifacts or unregulated signal amplification when processing the samples. As shown in Figure 6(a) (left panel), the transcription level of Mash-1 was increased in response to bFGF. The protein levels of Mash-1 were significantly emerging and increased after 24 h, which were observed as demonstrated by Western blot (Figure 6(a), right panel). Full unedited western blot images are supplied in Supplemental data 2. The expressions of neuronal-related genes, including GAP43 and Hes5, were also detected after bFGF treatment, which are also upregulated, but later than Mash-1 at mRNA levels (Figure 6(a)), indicating that Mash-1 may play an initiating role in driving neuronal differentiation of BMSCs. We also used immunofluorescence staining to test Mash1. The results showed that BMSCs treated with bFGF after 72 h exhibited high expression of Mash1, and neurogenesis-related markers, including GAP43 and Hes5, were also expressed on these cells (Figure 6(b)). To determine the proneuronal effect of Mash-1 in BMSCs, we cloned and transfected Mash-1 into mouse BMSCs. After transfection, Mash-1-overexpressing BMSCs exhibited neuron-like morphology, which was evidenced by elongated neurites and cell body shrinkages (Figure 6(c)). Biochemical analysis revealed a higher expression of neuronal marker, NSE, in Mash-1-overexpressing BMSCs (Figure 6(d)). NSE has neurotrophic and neuroprotective properties on a broad spectrum in central nervous system neurons. We also detected the expression of synaptic-associated genes, including SV2a and SNAP-25 (Figure 6(e)). SV2a is an essential vesicle membrane protein expressed in virtually all synapses and could serve as a suitable target for synaptic density. SNAP-25 is one of the N-ethylmaleimide-sensitive fusion protein receptor, which involved in synaptic vesicle docking and subsequent fusion with the target membrane. The data show SV2a and SNAP-25 were highly induced in Mash-1-overexpressing BMSCs, indicated a potential synaptic capability in these cells. All these results combined with our earlier observation suggest that Mash-1 may be a crucial proneuronal differentiation factor upon bFGF treatment.

## 4. Discussion

BMSCs were demonstrated to exhibit neural phenotypes both *in vivo* and *in vitro* under favourable conditions. Neural properties of BMSC generated cells, in most cases, were determined by measuring the expression of a few neural markers, which, in our opinion, would not reflect completely the phenotypes of neural traits [11] and leave the neuronal differentiation mechanisms much to our own imagination. In order to get a global pattern of gene expression changes in neuronal differentiation of BMSC, we investigated the kinetic changes of gene profiles of neuronal differentiated mouse BMSCs with the employment of cDNA microarray analysis. Our study revealed a strong inclination to upregulation of neural genes with various neural functions and properties. Interestingly, we further demonstrated that the Notch signaling molecule, Mash-1, plays an important role in driving BMSCs into adopting neuronal phenotype. These observations suggest that neuronal differentiation of BMSCs might mimic or undertake certain conserved neuronal differentiation pathways.

The powerful analytical tool of microarray analysis has been used to determine the neural properties of bFGF differentiated BMSCs. Mori et al. reported the first neural gene profiles by using immortalized human BMSCs [13] which was adopted by Yamaguchi in further determining the neural gene expression [14]. However, both of them used RNAs extracted from cells differentiated for more than a week as their templates, and Yamaguchi had even been using a series of chemicals including DMSO and beta-mercaptoethanol, to differentiate BMSCs, which was strictly argued as being a stress-induced response [10, 65]. Moreover, they covered only a small pool of genes in their studies where most of the neural genes identified were neural markers or molecules expressed in the mature/differentiated cell. In our study, we used bFGF alone to induce mouse BMSC neuronal differentiation at around 2-3 days, and the differentiated cells acquired electrophysiological properties [15]. The obvious upregulations of wide range neural genes suggest that neuronal differentiated BMSCs can adopt many neural phenotypes under physiological condition and not merely on neural marker expressions [11].

Neuronal differentiation of BMSCs, especially induced with various chemicals, was challenged as being a direct stress-induced response [65], and the cellular neurite-like processes of neuronal differentiated BMSCs in most studies were demonstrated to be formed by cytoplasm shrinkage, rather than the growth of neurites [10]. However, in our study, many axonal growths related genes such as Netrin-1 and Dyncine were obviously upregulated from 4 hours treatment by bFGF onwards. The high levels of expression of these genes clearly indicate that the neurite-like cell processes as observed are that of being an active protrusion and not simply a cell shrinkage phenomena as argued. Moreover, a large number of genes, including ion transportation, neurotransmitters, and peptide signaling such as GABA receptor, complexin, and opioid receptor, were also upregulated. In agreement to our previous finding, neuronal cells from bFGF-treated BMSCs exhibit electrophysiological properties [15], completing with a wide range of expression of neural genes, and it is clear that this indicated BMSCs could acquire more complete neuronal traits under physiological stimulation.

Studies on the development of the nervous system of *Drosophila* demonstrated that Notch receptor initiated signaling pathways play a crucial role in neuroblast generations, of which several groups of genes, including the bHLH family, are induced to regulate neural determination and differentiation [66]. In Drosophila, bFGF signaling pathway has been demonstrated to interact with the Notch signaling pathway in various cell types [67]. All the Notch pathway genes are preserved in vertebrates and expressed in nervous system and others [26]. The initiation and differentiation of BMSCs into neuronal cells by physiological factors were clearly involved in several neurogenesis pathways which are well documented in the development of *Drosophila* and neural stem cell differentiation. Though a close comparison to the Drosophila model could be drawn, distinctions of BMSC neuronal differentiation from that observed in Drosophila should still be addressed.

In BMSCs, the intracellular domain of Notch (NICD) was shown recently to be capable of inducing BMSC neuronal differentiation. This function was further demonstrated to be coordinated with bFGF [9]. Interestingly, bFGF was shown to promote the activity of Notch pathway in embryonic neuroepithelial precursor cells to inhibit differentiation [67]. The components of the Notch signaling pathways can be influenced by bFGF to affect the oligodendrocyte differentiation [68]. However, the mechanisms of the cross-talk between Notch and bFGF signaling pathways are need further identified. Adopting screening by microarray, we observed an astounding upregulation of Mash-1, the vertebrate homolog of bHLH transcription factor, which was also known as achaete-scute complex homolog-1 and shown to control the generation of progenitors for the central nervous system and external sensory organs in the peripheral nervous system [69, 70]. Previous study has delineated transcription factors Mash-1 and Prox-1 played a role in early steps during differentiation of neural stem cells in the developing central nervous system [48]. Mash1 has an important role in Notch signaling and the differentiation of neurons [71]. When we overexpressed Mash-1, BMSCs exhibited similar neuronal structures as differentiated by bFGF (Figure 6). These results suggested that Mash-1 could probably be the key factor to genes regulated by bFGF responsible for driving BMSC differentiation to neuronal cells. Similarly as our studies, Wang et al. show that under conditions of differentiation, Mash1-overexpressing BMSCs exhibit an increased expression of neuronal markers and a greater degree of neuronal morphology compared to control (non-Mash1-overexpressing cells) [72]. Xu et al. also indicated that overexpression of Mash-1 gene in promoting differentiation of mouse embryonic stem cells into neural cells [49]. Additionally studies showed Mash1-dependent Notch signaling pathway also regulates GABAergic neuron-like differentiation of BMSCs [50, 73].

Given so abundant investigations and reviews, various biomarkers of neuronal cells in characteristics were applied. Thus, it is necessary to apply multiple markers to the analysis cell type. In this work, we used these markers to identify the bFGF-induced neuronal differentiation of BMSCs. Gathering the analyzed data, we catch many markers were established in the development of bFGF-induced neurogenesis. While more neural markers may allow better reflection of neural traits, others believe that it is also true that science seeks to synthesize the identification of biological processes with fewer markers. However, it is difficult, using a single marker, to identify a single cell type exclusively. This work meets both ends, and Mash-1 is very useful gene for this purpose to identify the neuronal origin, which invokes to give a conceptual vision to the study of the stem cells and regenerative medicine.

In conclusion, our work reveals that the cross-talk is through the activation of downstream Notch components though the detail mechanisms of the activation of Mash-1 by bFGF. Concerns over the level of initiation, determination, and differentiation of BMSCs to proneuronal cells as induced by bFGF should be further analyzed. It is further noted that in this study, bFGF-induced neuronal differentiation of BMSCs opened a window of opportunity to studying the underlying mechanisms involved in the complex process of neuronal differentiation and maturation at physiological condition of BMSC neurogenesis. The genetic engineering of BMSCs will be a useful avenue for obtaining neuron-like donor cells for the treatment of neurological disorders.

## Figures and Tables

**Table 1:** 

Fold			Gene name	Accession number
4 h	24 h	72 h		
Early upregulated neuronal genes				
9.6	4.0	3.0	Synaptic vesicle glycoprotein 2a	NM_022030
5.9	3.7	3.4	Chemokine (C-C motif) receptor 1	NM_009912
5.5	2.1	1.6	Synaptosomal-associated protein 25	NM_011428
5.3	1.1	1.0	Gamma-aminobutyric acid receptor	NM_008076
Continuous upregulated neuronal genes				
4.4	6.3	11.0	Semaphorin 5A	NM_009154
2.0	7.4	8.2	P2-activated kinase 1	NM_011035
0.9	4.7	7.5	Putative neuronal cell adhesion molecule	AK051027
3.7	5.2	6.1	Semaphorin 6A	NM_018744

**Table 2:** 

Fold			Gene name	Accession number
4 h	24 h	72 h		
Neural development				
1.7	1.1	1.9	Syntrophin, acidic 1	NM_009228
2.1	3.0	0.8	Nestin	NM_016701
2.2	1.9	2.3	Empty spiracles homolog 1	X68881
0.9	2.4	5.8	Sema domain, immunoglobulin domain	NM_013657
2.3	1.5	4.5	Fibroblast growth factor receptor 1	NM_010206
1.6	1.5	2.2	Chordin	NM_009893
2.0	1.3	1.4	Sema domain, seven thrombospondin repeats	NM_013661
Transcription factor				
4.5	2.3	8.5	Achaete-scute complex homolog-like 1	NM_008553
4.4	1.9	9.9	Hairy and enhancer of split 5	NM_010419
1.4	1.5	3.2	Neuronal PAS domain protein 3	NM_013780
1.1	0.7	2.4	Neuronal PAS domain protein 2	NM_008719
3.1	2.1	1.4	Neurogenic differentiation 4	NM_007501
0.9	1.8	5.9	Ets variant gene 1	NM_007960
3.2	1.9	4.6	v-maf muculoaponeurotic fibrodarcoma oncogene	NM_010756
Axonal				
2.0	1.4	1.7	Kinesin family member 5C	NM_008449
2.0	2.2	2.2	Reticulon 4 receptor	NM_022982
1.2	1.6	2.7	Dynein, axonemal, light chain 4	NM_017470
2.1	1.8	0.5	Ephrin B1	NM_010110
1.4	1.1	2.1	Slit homolog 1	AF144627
3.1	3.3	2.8	Dynein, axonemal, heavy chain 10	Z83812
21.8	15.2	22.1	Netrin 1	NM_008744
Neurotransmitter				
0.1	3.6	5.1	Ral guanine nucleotide dissociation stimulator	NM_009058
1.0	2.3	0.8	Amyloid beta precursor protein-binding	AF020313
1.4	1.2	3.2	Syntaxin 4A	NM_009294
1.4	1.0	2.2	Syntaxin 3	D29797
3.0	1.3	5.3	Complexin 1	NM_007756
3.7	5.3	3.7	Suppressor of cytokine signaling 2	NM_007706
2.4	1.4	2.3	Solute carrier family 6 (neurotransmitter transporter)	AK036136
Ion transporter				
0.7	2.4	1.8	Gamma-aminobutyric acid receptor, alpha 4	AK013727
3.2	3.3	5.1	Gamma-aminobutyric acid receptor, alpha 6	NM_008068
1.8	1.2	1.9	Sema domain, transmembrane domain	NM_013662
2.0	1.3	1.4	Sema domain, seven thrombospondin repeats	NM_013661
2.8	4.0	3.7	Cholinergic receptor, nicotinic, alpha polypeptide 7	NM_007390
1.3	1.6	1.7	Cholinergic receptor, nicotinic, beta polypeptide 1	NM_009601
G-protein				
0.8	2.6	2.2	CD97 antigen	NM_011925
2.8	1.5	4.5	Opioid receptor, mu 1	NM_011013
0.6	2.2	1.0	G-protein-coupled receptor 56	NM_018882
2.9	1.1	1.6	Chemokine receptor 3	NM_009914
3.0	3.1	3.9	G-protein-coupled receptor 50	NM_010340
3.8	2.0	4.1	G-protein-coupled receptor 54	NM_053244
Others				
0.9	4.7	7.5	Putative neuronal cell adhesion molecule	AK051027
1.0	2.3	0.8	Amyloid beta precursor protein-binding	AF020313
2.3	1.7	1.7	EF hand calcium binding protein 2	NM_054095
1.4	1.0	2.7	Activity-dependent neuroprotective protein	NM_009628
2.7	2.1	4.7	Brain glycogen phosphorylase	NM_153781.1
1.7	2.4	3.2	Phospholipase C, epsilon 1	BC025027

## Data Availability

The data used to support the findings of this study are included within the article.

## Supplementary Materials

Supplementary Materials Full unedited western blot images of Figures 3(c) and 6(a) data are supplied in Supplementary materials.

## References

[1] Prockop D. J. (1997). Marrow stromal cells as stem cells for nonhematopoietic tissues. *Science*.

[2] Bianco P., Riminucci M., Gronthos S., Robey P. G. (2001). Bone marrow stromal stem cells: nature, biology, and potential applications. *Stem Cells*.

[3] Kopen G. C., Prockop D. J., Phinney D. G. (1999). Marrow stromal cells migrate throughout forebrain and cerebellum, and they differentiate into astrocytes after injection into neonatal mouse brains. *Proceedings of the National Academy of Sciences of the United States of America*.

[4] Ide C., Nakai Y., Nakano N. (2010). Bone marrow stromal cell transplantation for treatment of sub-acute spinal cord injury in the rat. *Brain Research*.

[5] Chopp M., Zhang X. H., Li Y. (2000). Spinal cord injury in rat: treatment with bone marrow stromal cell transplantation. *Neuroreport*.

[6] Corti S., Locatelli F., Papadimitriou D., Strazzer S., Comi G. P. (2004). Somatic stem cell research for neural repair: current evidence and emerging perspectives. *Journal of Cellular and Molecular Medicine*.

[7] Corti S., Locatelli F., Strazzer S., Guglieri M., Comi G. P. (2003). Neuronal generation from somatic stem cells: current knowledge and perspectives on the treatment of acquired and degenerative central nervous system disorders. *Current Gene Therapy*.

[8] Sanchez-Ramos J. R. (2002). Neural cells derived from adult bone marrow and umbilical cord blood. *Journal of Neuroscience Research*.

[9] Dezawa M. (2005). Specific induction of neurons and Schwann cells from bone marrow stromal cells and application to neurodegenerative diseases. *No Shinkei Geka*.

[10] Neuhuber B., Gallo G., Howard L., Kostura L., Mackay A., Fischer I. (2004). Reevaluation of in vitro differentiation protocols for bone marrow stromal cells: disruption of actin cytoskeleton induces rapid morphological changes and mimics neuronal phenotype. *Journal of Neuroscience Research*.

[11] Raedt R., Pinxteren J., van Dycke A. (2007). Differentiation assays of bone marrow-derived Multipotent Adult Progenitor Cell (MAPC)-like cells towards neural cells cannot depend on morphology and a limited set of neural markers. *Experimental Neurology*.

[12] Egusa H., Schweizer F. E., Wang C. C., Matsuka Y., Nishimura I. (2005). Neuronal differentiation of bone marrow-derived stromal stem cells involves suppression of discordant phenotypes through gene silencing. *The Journal of Biological Chemistry*.

[13] Mori T., Kiyono T., Imabayashi H. (2005). Combination of hTERT and bmi-1, E6, or E7 induces prolongation of the life span of bone marrow stromal cells from an elderly donor without affecting their neurogenic potential. *Molecular and Cellular Biology*.

[14] Yamaguchi S., Kuroda S., Kobayashi H. (2006). The effects of neuronal induction on gene expression profile in bone marrow stromal cells (BMSC)--a preliminary study using microarray analysis. *Brain Research*.

[15] Yang H., Xia Y., Lu S. Q., Soong T. W., Feng Z. W. (2008). Basic fibroblast growth factor-induced neuronal differentiation of mouse bone marrow stromal cells requires FGFR-1, MAPK/ERK, and transcription factor AP-1. *The Journal of Biological Chemistry*.

[16] Woodbury D., Schwarz E. J., Prockop D. J., Black I. B. (2000). Adult rat and human bone marrow stromal cells differentiate into neurons. *Journal of Neuroscience Research*.

[17] Black I. B., Woodbury D. (2001). Adult rat and human bone marrow stromal stem cells differentiate into neurons. *Blood Cells, Molecules & Diseases*.

[18] Wilson S. I., Graziano E., Harland R., Jessell T. M., Edlund T. (2000). An early requirement for FGF signalling in the acquisition of neural cell fate in the chick embryo. *Current Biology*.

[19] Henrique D., Tyler D., Kintner C. (1997). cash4, a novel achaete-scute homolog induced by Hensen’s node during generation of the posterior nervous system. *Genes & Development*.

[20] Taetzsch T., Brayman V. L., Valdez G. (2018). FGF binding proteins (FGFBPs): modulators of FGF signaling in the developing, adult, and stressed nervous system. *Biochimica et Biophysica Acta - Molecular Basis of Disease*.

[21] LIU Y., YI X. C., GUO G. (2014). Basic fibroblast growth factor increases the transplantation-mediated therapeutic effect of bone mesenchymal stem cells following traumatic brain injury. *Molecular Medicine Reports*.

[22] Chiba S., Kurokawa M. S., Yoshikawa H. (2005). Noggin and basic FGF were implicated in forebrain fate and caudal fate, respectively, of the neural tube-like structures emerging in mouse ES cell culture. *Experimental Brain Research*.

[23] Sakaguchi D. S., Janick L. M., Reh T. A. (1997). Basic fibroblast growth factor (FGF-2) induced transdifferentiation of retinal pigment epithelium: generation of retinal neurons and glia. *Developmental Dynamics*.

[24] Wu Y., Wang Z., Cai P. (2018). Dual delivery of bFGF- and NGF-binding coacervate confers neuroprotection by promoting neuronal proliferation. *Cellular Physiology and Biochemistry*.

[25] Knust E., Campos-Ortega J. A. (1989). The molecular genetics of early neurogenesis inDrosophila melanogaster. *BioEssays*.

[26] Zhang R., Engler A., Taylor V. (2018). Notch: an interactive player in neurogenesis and disease. *Cell and Tissue Research*.

[27] Engler A., Zhang R., Taylor V. (2018). Notch and neurogenesis. *Advances in Experimental Medicine and Biology*.

[28] Richards G. S., Rentzsch F. (2015). Regulation of Nematostella neural progenitors by SoxB, Notch and bHLH genes. *Development*.

[29] Brunet J. F., Ghysen A. (1999). Deconstructing cell determination: proneural genes and neuronal identity. *BioEssays*.

[30] Gaiano N., Fishell G. (2002). The role of notch in promoting glial and neural stem cell fates. *Annual Review of Neuroscience*.

[31] de la Pompa J. L., Wakeham A., Correia K. M. (1997). Conservation of the Notch signalling pathway in mammalian neurogenesis. *Development*.

[32] Zhang L., Su P., Xu C., Yang J., Yu W., Huang D. (2010). Chondrogenic differentiation of human mesenchymal stem cells: a comparison between micromass and pellet culture systems. *Biotechnology Letters*.

[33] Banfi A., Bianchi G., Notaro R., Luzzatto L., Cancedda R., Quarto R. (2002). Replicative aging and gene expression in long-term cultures of human bone marrow stromal cells. *Tissue Engineering*.

[34] Baustian C., Hanley S., Ceredig R. (2015). Isolation, selection and culture methods to enhance clonogenicity of mouse bone marrow derived mesenchymal stromal cell precursors. *Stem Cell Research & Therapy*.

[35] Meirelles L. S., Nardi N. B. (2003). Murine marrow-derived mesenchymal stem cell: isolation,in vitroexpansion, and characterization. *British Journal of Haematology*.

[36] Sekiya I., Larson B. L., Smith J. R., Pochampally R., Cui J.&;. G., Prockop D. J. (2002). Expansion of human adult stem cells from bone marrow stroma: conditions that maximize the yields of early progenitors and evaluate their quality. *Stem Cells*.

[37] Loscher W., Gillard M., Sands Z. A., Kaminski R. M., Klitgaard H. (2016). Synaptic vesicle glycoprotein 2A ligands in the treatment of epilepsy and beyond. *CNS Drugs*.

[38] Halks-Miller M., Schroeder M. L., Haroutunian V. (2003). CCR1 is an early and specific marker of Alzheimer’s disease. *Annals of Neurology*.

[39] Noor A., Zahid S. (2016). A review of the role of synaptosomal-associated protein 25 (SNAP-25) in neurological disorders. *The International Journal of Neuroscience*.

[40] Brohan J., Goudra B. G. (2017). The role of GABA receptor agonists in anesthesia and sedation. *CNS Drugs*.

[41] Hilario J. D., Rodino-Klapac L. R., Wang C., Beattie C. E. (2009). Semaphorin 5A is a bifunctional axon guidance cue for axial motoneurons in vivo. *Developmental Biology*.

[42] Lee A. R., Ko K. W., Lee H., Yoon Y. S., Song M. R., Park C. S. (2016). Putative cell adhesion membrane protein Vstm5 regulates neuronal morphology and migration in the central nervous system. *The Journal of Neuroscience*.

[43] Wei Y., Gong J., Xu Z. (2015). Nrf2 in ischemic neurons promotes retinal vascular regeneration through regulation of semaphorin 6A. *Proceedings of the National Academy of Sciences of the United States of America*.

[44] Lendahl U., Zimmerman L. B., McKay R. D. G. (1990). CNS stem cells express a new class of intermediate filament protein. *Cell*.

[45] Sasai Y., Lu B., Steinbeisser H., De Robertis E. M. (1995). Regulation of neural induction by the Chd and Bmp-4 antagonistic patterning signals in Xenopus. *Nature*.

[46] Mayor R., Guerrero N., Martinez C. (1997). Role of FGF and noggin in neural crest induction. *Developmental Biology*.

[47] Bertrand N., Castro D. S., Guillemot F. (2002). Proneural genes and the specification of neural cell types. *Nature Reviews. Neuroscience*.

[48] Torii Ma, Matsuzaki F., Osumi N. (1999). Transcription factors Mash-1 and Prox-1 delineate early steps in differentiation of neural stem cells in the developing central nervous system. *Development*.

[49] Xu L., Ding D., Li X., Shi Q., Wang Y., Zhou C. (2015). Over expression of Mash-1 gene in promoting differentiation of mouse embryonic stem cells into neural cells. *Zhongguo Xiu Fu Chong Jian Wai Ke Za Zhi*.

[50] Long Q., Luo Q., Wang K., Bates A., Shetty A. K. (2017). Mash1-dependent Notch signaling pathway regulates GABAergic neuron-like differentiation from bone marrow-derived mesenchymal stem cells. *Aging and Disease*.

[51] Liu Z. H., Dai X. M., Du B. (2015). Hes1: a key role in stemness, metastasis and multidrug resistance. *Cancer Biology & Therapy*.

[52] Kageyama R., Ohtsuka T. (1999). The Notch-Hes pathway in mammalian neural development. *Cell Research*.

[53] Sommer L., Ma Q., Anderson D. J. (1996). Neurogenins, a novel family of atonal-related bHLH transcription factors, are putative mammalian neuronal determination genes that reveal progenitor cell heterogeneity in the developing CNS and PNS. *Molecular and Cellular Neurosciences*.

[54] Schäck L., Budde S., Lenarz T. (2016). Induction of neuronal-like phenotype in human mesenchymal stem cells by overexpression of neurogenin1 and treatment with neurotrophins. *Tissue & Cell*.

[55] Pieper A. A., Wu X., Han T. W. (2005). The neuronal PAS domain protein 3 transcription factor controls FGF-mediated adult hippocampal neurogenesis in mice. *Proceedings of the National Academy of Sciences of the United States of America*.

[56] Porter M. E. (1996). Axonemal dyneins: assembly, organization, and regulation. *Current Opinion in Cell Biology*.

[57] Serafini T., Kennedy T. E., Gaiko M. J., Mirzayan C., Jessell T. M., Tessier-Lavigne M. (1994). The netrins define a family of axon outgrowth-promoting proteins homologous to C. elegans UNC-6. *Cell*.

[58] McMahon H. T., Missler M., Li C., Südhof T. C. (1995). Complexins: cytosolic proteins that regulate SNAP receptor function. *Cell*.

[59] Bennett M. K., Calakos N., Scheller R. H. (1992). Syntaxin: a synaptic protein implicated in docking of synaptic vesicles at presynaptic active zones. *Science*.

[60] Gassmann M., Bettler B. (2012). Regulation of neuronal GABA(B) receptor functions by subunit composition. *Nature Reviews. Neuroscience*.

[61] Pasterkamp R. J. (2012). Getting neural circuits into shape with semaphorins. *Nature Reviews. Neuroscience*.

[62] Kauffman A. S., Park J. H., McPhie-Lalmansingh A. A. (2007). The kisspeptin receptor GPR54 is required for sexual differentiation of the brain and behavior. *The Journal of Neuroscience*.

[63] Liu M., Parker R. M., Darby K. (1999). GPR56, a novel secretin-like human G-protein-coupled receptor gene. *Genomics*.

[64] Rossi G., Pan Y. X., Cheng J., Pasternak G. W. (1994). Blockade of morphine analgesia by an antisense oligodeoxynucleotide against the mu receptor. *Life Sciences*.

[65] Lu P., Blesch A., Tuszynski M. H. (2004). Induction of bone marrow stromal cells to neurons: differentiation, transdifferentiation, or artifact?. *Journal of Neuroscience Research*.

[66] San-Juan B. P., Baonza A. (2011). The bHLH factor deadpan is a direct target of Notch signaling and regulates neuroblast self-renewal in Drosophila. *Developmental Biology*.

[67] Faux C. H., Turnley A. M., Epa R., Cappai R., Bartlett P. F. (2001). Interactions between fibroblast growth factors and Notch regulate neuronal differentiation. *The Journal of Neuroscience*.

[68] Zhou Y. X., Armstrong R. C. (2007). Interaction of fibroblast growth factor 2 (FGF2) and notch signaling components in inhibition of oligodendrocyte progenitor (OP) differentiation. *Neuroscience Letters*.

[69] Battiste J., Helms A. W., Kim E. J. (2007). Ascl1 defines sequentially generated lineage-restricted neuronal and oligodendrocyte precursor cells in the spinal cord. *Development*.

[70] Sugimori M., Nagao M., Bertrand N., Parras C. M., Guillemot F., Nakafuku M. (2007). Combinatorial actions of patterning and HLH transcription factors in the spatiotemporal control of neurogenesis and gliogenesis in the developing spinal cord. *Development*.

[71] Serre A., Snyder E. Y., Mallet J., Buchet D. (2012). Overexpression of basic helix-loop-helix transcription factors enhances neuronal differentiation of fetal human neural progenitor cells in various ways. *Stem Cells and Development*.

[72] Wang K., Long Q., Jia C. (2013). Over-expression of Mash1 improves the GABAergic differentiation of bone marrow mesenchymal stem cells in vitro. *Brain Research Bulletin*.

[73] Oishi K., Watatani K., Itoh Y. (2009). Selective induction of neocortical GABAergic neurons by the PDK1-Akt pathway through activation of Mash1. *Proceedings of the National Academy of Sciences of the United States of America*.

