# Supplementary material for: Gene Profiles in the Early Stage of Neuronal Differentiation of Mouse Bone Marrow Stromal Cells Induced by Basic Fibroblast Growth Factor
Source: Stem Cells Int. 2020 Dec 24;2020:8857057. doi: 10.1155/2020/8857057 (PMC7775150; doi:10.1155/2020/8857057)
Supplement: Supplementary Materials — Full unedited western blot images of Figures 3(c) and 6(a) data are supplied in Supplementary materials. [file 8857057.f1.pdf]

Supplemental data 1

β-actin

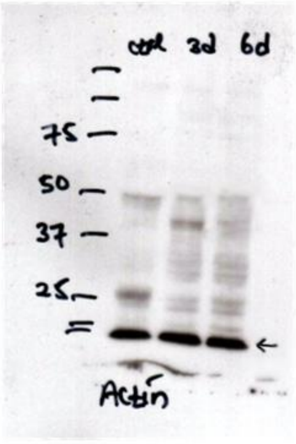

ChAT

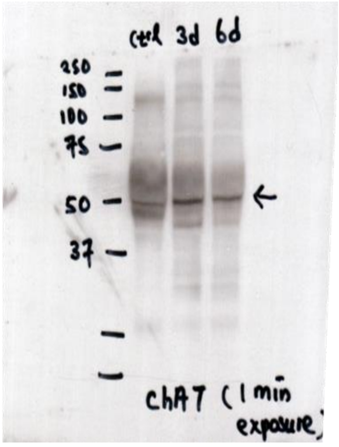

GAP43

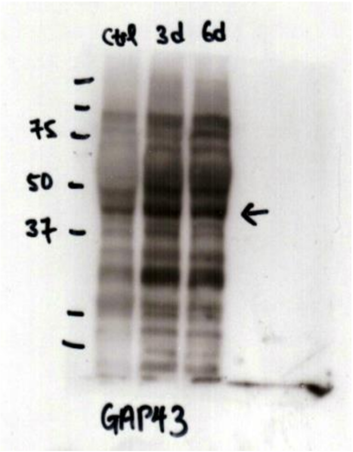

NSE

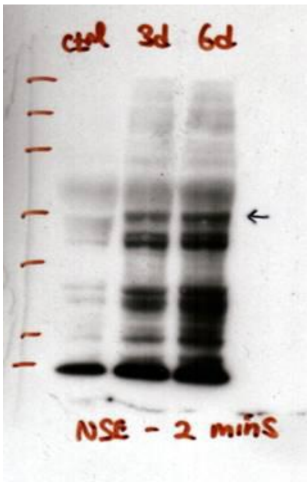

Tau

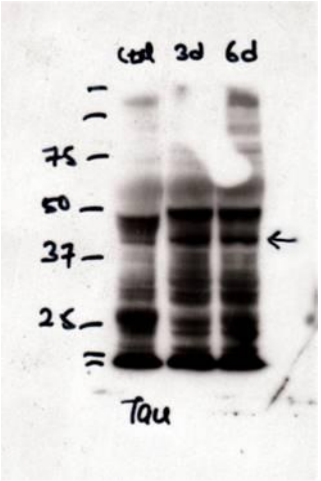

Full unedited western blot images data in Figure 3C.

Supplemental data 2

β-actin

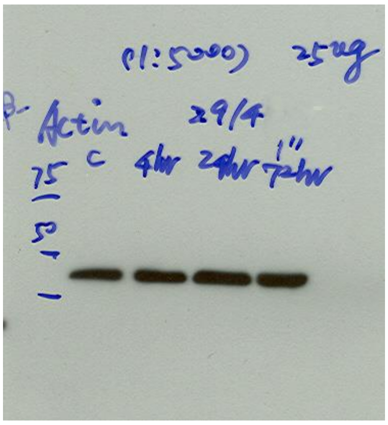

GAP43

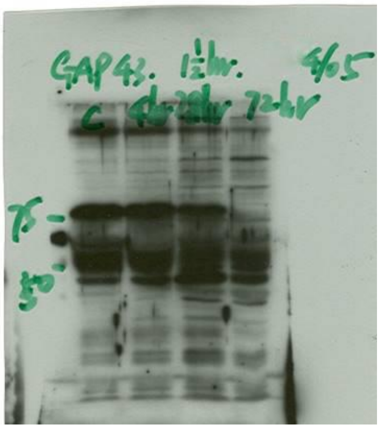

Mash-1

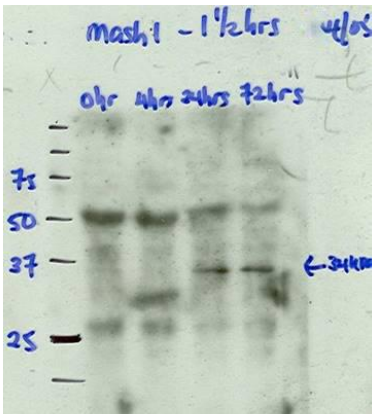

Hes5

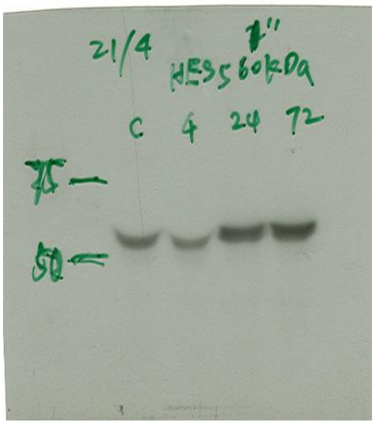

Full unedited western blot images data in Figure 6A.
